# Public perceptions of climate tipping points

**DOI:** 10.1177/09636625231177820

**Published:** 2023-06-28

**Authors:** Rob Bellamy

**Affiliations:** The University of Manchester, UK

**Keywords:** climate tipping points, cultural cognition, Hothouse Earth, public perception, risk, tipping cascades

## Abstract

Coverage of climate tipping points has rapidly increased over the past 20 years. Despite this upsurge, there has been precious little research into how the public perceives these abrupt and/or irreversible large-scale risks. This article provides a nationally representative view on public perceptions of climate tipping points and possible societal responses to them (*n* = 1773). Developing a mixed-methods survey with cultural cognition theory, it shows that awareness among the British public is low. The public is doubtful about the future effectiveness of humanity’s response to climate change in general, and significantly more doubtful about its response to tipping points specifically. Significantly more people with an egalitarian worldview judge tipping points likely to be crossed and to be a significant threat to humanity. All possible societal responses received strong support. The article ends by considering the prospects for ‘cultural tipping elements’ to tip support for climate policies across divergent cultural worldviews.

## 1. Introduction

**Figure 1.**
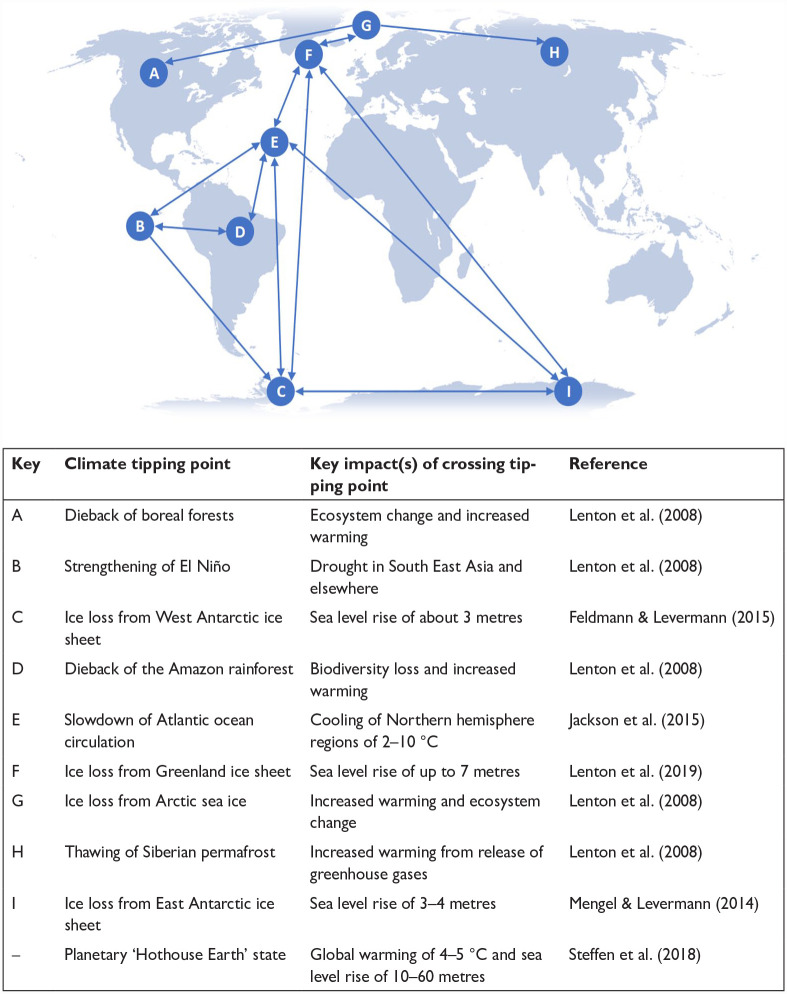
Global map of potential tipping cascades. Arrows show potential interactions among tipping elements based on expert elicitation that could generate cascades (see Lenton et al., 2019; Steffen et al., 2018).

The Sixth Assessment Report of the Intergovernmental Panel on Climate Change (IPCC) has concluded that climate tipping points – critical thresholds at which a small perturbation can abruptly and/or irreversibly alter the state or development of a system – cannot be ruled out (IPCC, 2021). Tipping points are thought to exist for a number of large-scale components of the Earth’s climate system, also called tipping elements (Lenton et al., 2008, 2019; McKay et al., 2022; Steffen et al., 2018) (Figure 1). These include dieback of the boreal and Amazon forests, ice loss from Arctic sea ice as well as the Greenland and Antarctic ice sheets, thawing of Siberian permafrost, strengthening of the El Niño Southern Oscillation and slowdown of the Atlantic Meridional Overturning Circulation (AMOC). It is now thought that certain climate tipping points could interact with one another to produce cascading effects (Lenton et al., 2019; Rocha et al., 2018). For example, ice loss from Arctic sea ice and the Greenland ice sheet could be driving a 15% slowdown in the AMOC through an influx of freshwater into the North Atlantic (Caesar et al., 2018). On a larger scale, a global cascade of tipping points could trigger a global tipping point to a new, less habitable, planetary ‘Hothouse Earth’ state (Steffen et al., 2018). Such events are thought to risk societal collapse or even human extinction as part of a ‘climate endgame’ (Kemp et al., 2022).

**Figure 2.**
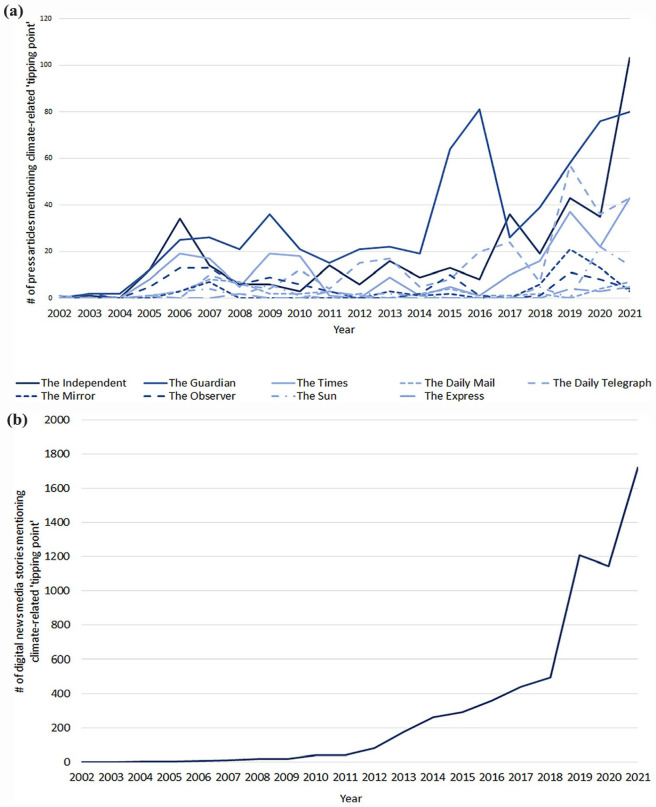
Annual trends in reporting climate-related tipping points in (a) UK national broadsheet and tabloid press and (b) global English-language digital news media. The full text of each item was searched in (a) via the LexisNexis database and in (b) via the MediaCloud database for instances of the phrase ‘tipping point’ and ‘climate change’ or ‘global warming’ or ‘global heating’ or ‘climate emergency’ or ‘climate crisis’ or ‘climate breakdown’ from 2002 to 2021 (years prior to this had no instances of climate-related tipping points and are excluded from this figure). For (a), politically centrist papers are coloured dark blue; left and centre-left papers are blue; and right and centre-right papers are light blue.

An analysis of annual trends in the coverage of climate tipping points using the LexisNexis and MediaCloud databases shows a rapid increase in both British print and global English-language online media over the past 20 years (Figure 2). An initial ‘tipping point trend’ in climate change communication was previously reported up until 2007 (Russill and Nyssa, 2009), but new analysis shows this was followed by a plateau before rapidly increasing again around the time of the 21st session of the Conference of the Parties (COP 21) in Paris in 2015. Despite this upsurge, beyond media analysis such as these and others (Antilla, 2010; Van der Hel et al., 2018), there has been precious little research into how the public perceives climate tipping points. And yet, understanding public perception will be critical to developing effective ways of communicating the risks of tipping points and policy responses that account for different societal values and preferences (Whitmarsh and Capstick, 2018). In one study on 404 moviegoers, responses to the film *The Day After Tomorrow*, which depicts a shutdown of the AMOC, showed an increase in concern about climate change but a decrease in the perceived likelihood and temporal proximity of abrupt changes (Lowe et al., 2006). In a second study on 287 respondents at the University of East Anglia, climate tipping points were found to be most concerning among those with egalitarian values and produced a strong fatalistic narrative of helplessness, societal collapse and catastrophe (Bellamy and Hulme, 2011). In contrast, a third study on 381 respondents to an online experiment showed that nonlinear portrayals of climate change do not lead to perceptions of climate change being less controllable or more catastrophic (Formanski et al., 2022).

This article asks, how does the public perceive the risks of climate tipping points and what are their preferences in responding to them? To answer this question, it measures key variables relevant to the effective risk management of climate tipping points: *awareness* of the risks; perceptions of the *likelihood* and *threat* of those risks; and the perceived *effectiveness* of and *preferences* for responding to the risks. In doing so, the article seeks to build upon earlier research in several ways. First, it provides a large-scale, nationally representative view on public perceptions of climate tipping points (*n* = 1773). Second, it examines perceptions of a planetary Hothouse Earth state, dieback of the boreal and Amazon forests, and ice loss from the East Antarctic ice sheet, as well as providing an updated view on perceptions of Arctic sea ice and the Greenland and West Antarctic ice sheets, the AMOC, El Niño and Siberian permafrost. Third, it examines preferences among the full range of possible societal responses to climate tipping points – from energy conservation and efficiency to low carbon energy, carbon dioxide removal, solar geoengineering and adaptation. Fourth, it develops a mixed-methods survey with both quantitative and qualitative data to provide insights into both the significance and meaning of public perceptions.

In developing this method, the article applies cultural cognition (Kahan, 2012), the latest conception of the cultural theory of risk applied in earlier research (Bellamy and Hulme, 2011), to understand the socio-cultural basis for people’s perceptions (Douglas and Wildavsky, 1982). Cultural cognition posits that individuals’ perceptions of risks and responses are shaped by the social groups of which they are part. In particular, it describes two crosscutting dimensions of sociality – individualism-collectivism and hierarchy-egalitarianism – which give rise to four distinctive worldviews. Evidence for the formation of perceptions consistent with these worldviews has been collected for a wide range of risks and explains variation better than other individual characteristics such as education, income, personality types and political ideology (Kahan, 2012). As individuals become more egalitarian and collectivist, they become more concerned about climate change and other environmental risks such as nuclear waste and air pollution. In contrast, as individuals become more hierarchical and individualist, they become less concerned.

## 2. Methods

A nationally representative sample of the British public (*n* = 1,773) was recruited through a specialist panel company to complete the survey (see Supplemental Material). The sample was developed to be representative of the country’s make-up in terms of age, gender, social grade (an occupationally based socio-economic classification produced by the UK Office for National Statistics that differentiates positions within labour markets and production units in terms of their typical employment relations), region and political orientation (as measured by past election vote and European Union referendum vote). In addition, data on level of education (coded using the European Survey version of the International Standard Classification of Education) and social media use (operationalised as whether or not respondents were active members of selected popular social media networks) were collected for use in the analysis. The survey consisted of three stages (see Supplemental Material).

The first stage sought to measure respondents’ cultural worldviews as described by cultural cognition. Cultural worldviews were measured using the short-form individualism-collectivism and hierarchy-egalitarianism scales (British subjects wording) developed by Kahan et al. (2015). The individualism-collectivism scale measured attitudes towards social arrangements that expect individuals to attain their own well-being without interference from society versus those that expect society to ensure collective welfare. The hierarchy-egalitarianism scale measured attitudes towards social arrangements that link authority to stratified social roles based on attributes such as gender, ethnicity and class. For each scale item, subjects indicated agreement or disagreement on a 7-point Likert-type scale. The cultural cognition scales allow us to plot the location of individuals on a cultural cognition map (see Supplemental Material). Accordingly, this was used to classify individuals into one of the four groups of cultural worldview as described by the cultural cognition thesis. Cronbach’s alpha coefficients showed very reliable internal consistencies for both the individualism-collectivism (α = .70) and the hierarchy-egalitarianism (α = .88) scales.

The second stage introduced respondents to the topic of climate tipping points and asked them which of the 10 selected climate tipping points they were aware of prior to taking the survey. The 10 climate tipping points were selected for their spatial diversity (including physical locations as well as terrestrial and marine environments, and hydrosphere, cryosphere and biosphere systems), their capacity for cascading interactions between one another (Lenton et al., 2019) and their scale (nine represented different Earth subsystems, and one – the planetary ‘Hothouse Earth’ state – represented the Earth system as a whole (Steffen et al., 2018)). Respondents were then provided with a world map of these climate tipping points and information on their key impacts if the tipping points were to be crossed. They were then asked to identify those climate tipping points they felt were likely to be crossed as a result of human activities and those they felt posed a serious threat to humanity.

The third stage of the survey turned to possible societal responses to climate tipping points and began by asking respondents how effectively they felt humanity would be able to respond relative to climate change in general on a 7-point Likert-type scale. The respondents were then introduced to the six broad options for tackling climate change (Caldeira et al., 2013) – energy conservation, energy efficiency, low carbon energy, carbon dioxide removal, solar geoengineering and adaptation – and asked about the extent to which they would support or oppose them on a 7-point Likert-type scale. Finally, they were asked to identify the one option they felt most strongly about in their appraisal (be it in support or opposition) and explain why they felt that way in a qualitative, open-ended question.

The subsequent quantitative data analysis utilised statistical tests described in the main text and for the qualitative data followed established procedures for inductive, semantic and constructionist thematic analysis whereby the author became familiar with the data, generated initial codes, searched for themes, reviewed themes, defined and named themes, and reported them (Braun and Clarke, 2006).

## 3. Results

### Awareness of climate tipping points

Prior to taking the survey, respondents were most aware of ice loss from Arctic sea ice (58.3% of respondents), dieback of the Amazon rainforest (54.1%) and ice loss from the Greenland ice sheet (49.5%). They were somewhat aware of thawing of the Siberian permafrost (35.6%), ice loss from the West Antarctic (32.8%) and East Antarctic ice sheets (32.4%) and strengthening of El Niño (28.9%). Respondents were least aware of the slowdown of the Atlantic ocean circulation and a Hothouse Earth state (19.6% each), and dieback of boreal forest (17.8%). A little more than a quarter had not heard of any of these climate tipping points prior to taking the survey (25.4%).

A series of Pearson’s χ^2^ tests revealed a number of statistically significant differences in awareness of climate tipping points between different groups of respondents (see Supplemental Material). There is evidence of a significant relationship between cultural worldview and awareness, with egalitarian collectivists being aware of significantly more climate tipping points than other worldviews. Male, older, more educated and higher social grade respondents are also aware of significantly more climate tipping points than women, younger, less educated and lower social grade respondents.

There was no relationship between the use of social media and awareness of climate tipping points.

### Risk perceptions of climate tipping points

Dieback of the Amazon rainforest was the climate tipping point most considered likely to be crossed as a result of human activities. Strengthening of El Niño, slowdown of the Atlantic ocean circulation and a Hothouse Earth state were the least considered likely to be crossed. Dieback of the Amazon rainforest was also the climate tipping point most considered a serious threat to humanity, closely followed by a Hothouse Earth state and ice loss from the Greenland ice sheet. Slowdown of the Atlantic ocean circulation was the least considered to be a serious threat.

**Figure 3.**
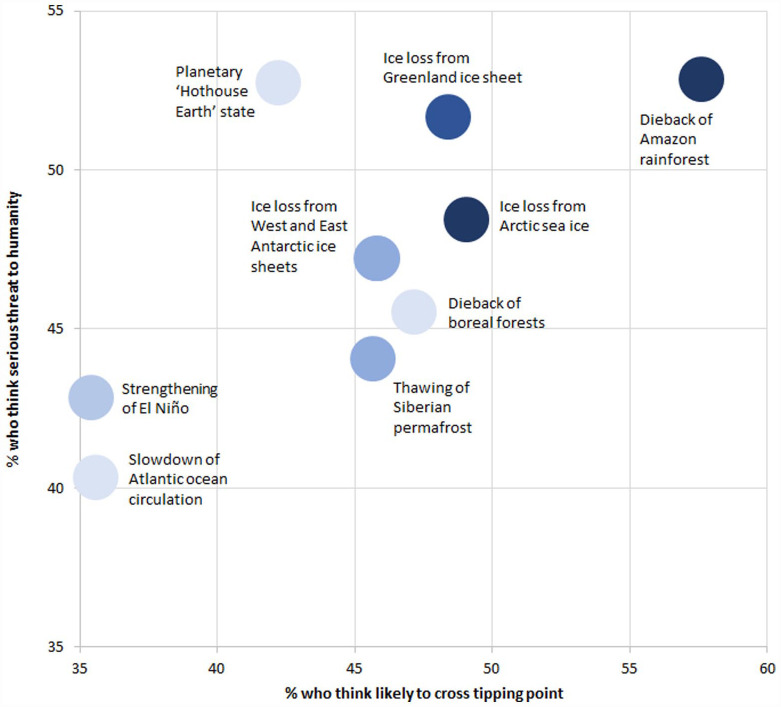
Risk perceptions of climate tipping points. Shading indicates the percentage of respondents who were aware of the corresponding climate tipping points prior to taking the survey, where the darkest shading indicates more than 50% awareness, dark indicates between 40% and 49%, moderate indicates between 30% and 39%, light indicates between 20% and 29%, and lightest indicates less than 20% awareness.

Taking these considerations of likelihood and impact together, the climate tipping points most considered to be a risk were dieback of the Amazon rainforest, ice loss from the Greenland ice sheet and ice loss from Arctic sea ice (Figure 3). In contrast, those least considered to be a risk were slowdown of the Atlantic ocean circulation and strengthening of El Niño. A Hothouse Earth state is an outlier in that it was relatively less considered a climate tipping point likely to be crossed, but among the most considered to be a serious threat to humanity.

A series of Pearson’s χ^2^ tests revealed a number of statistically significant differences in perceived risk of climate tipping points between different groups of respondents (see Supplemental Material). There is evidence of a significant relationship between cultural worldview and perceived likelihood of climate tipping points being crossed, with egalitarian collectivists and egalitarian individualists judging the likelihood to be significantly higher than other worldviews and hierarchical individualists judging it to be significantly lower. There is also evidence of a significant relationship between cultural worldview and perceived threat of climate tipping points, with egalitarian collectivists and egalitarian individualists judging the threat to be significantly higher than other worldviews and hierarchical individualists judging it to be significantly lower. Women and more educated respondents judge the likelihood of tipping points being crossed significantly higher than men and less educated respondents. Women and higher social grade respondents judge the threat of climate tipping points significantly higher than men and other social grades.

A minority of respondents judged the future effectiveness of humanity’s response to climate change in general (28.8%), climate tipping points specifically (20.0%) and a Hothouse Earth state specifically (19.9%) to range from fairly to extremely effective. The majority judged the future effectiveness of humanity’s response to climate change (46.1%), climate tipping points (49.2%) and a Hothouse Earth state (46.6%) to range from fairly to extremely ineffective. The remaining respondents indicated that they did not know about climate change in general (10.7%), climate tipping points (15.7%) and a Hothouse Earth state (19.5%), respectively. Following a Shapiro–Wilk test for normality of distribution, a nonparametric Wilcoxon’s signed-rank test revealed that perceived effectiveness was statistically significantly lower for climate tipping points (*Z* = –9.364, *p* < .001) and a Hothouse Earth state (*Z* = –8.217, *p* < .001) compared to climate change in general.

### Preferences for responding to climate tipping points

**Figure 4.**
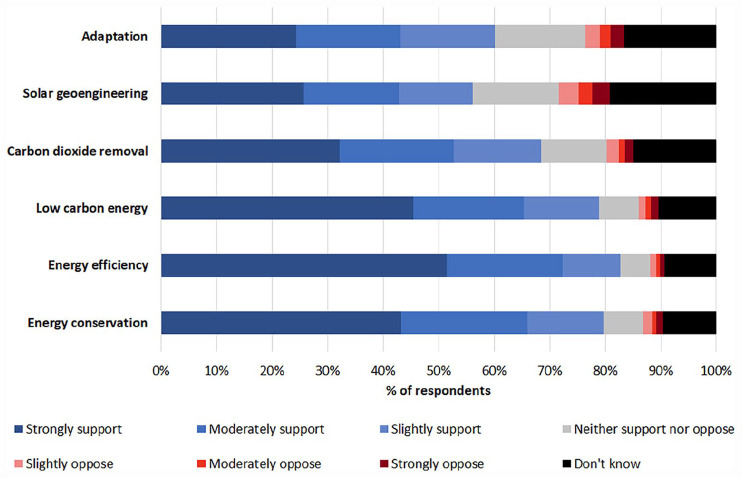
Support for different societal responses to climate tipping points.

In evaluating possible societal responses to climate tipping points, the majority of respondents expressed slight to strong support for all six broad options under consideration – energy efficiency (82.8%), energy conservation (79.7%), low carbon energy (78.8%), carbon dioxide removal (68.5%), adaptation (60.1%) and solar geoengineering (56.2%) (Figure 4).

Following a Shapiro–Wilk test for normality of distribution, a series of nonparametric analyses of variance were performed to test the differences between the mean levels of support under different cultural worldviews. A nonparametric Levene’s test showed homoscedasticity (i.e. groups having the same or similar variances, also called homogeneity of variance) in two of the samples, for which Kruskal–Wallis *H* tests were performed. For the remainder that showed heteroscedasticity (i.e. groups having different variances, also called heterogeneity of variance), a series of Mood’s median tests were performed (unlike the Kruskal–Wallis *H* test, the Mood’s median test does not assume homogeneity of variance).

**Figure 5.**
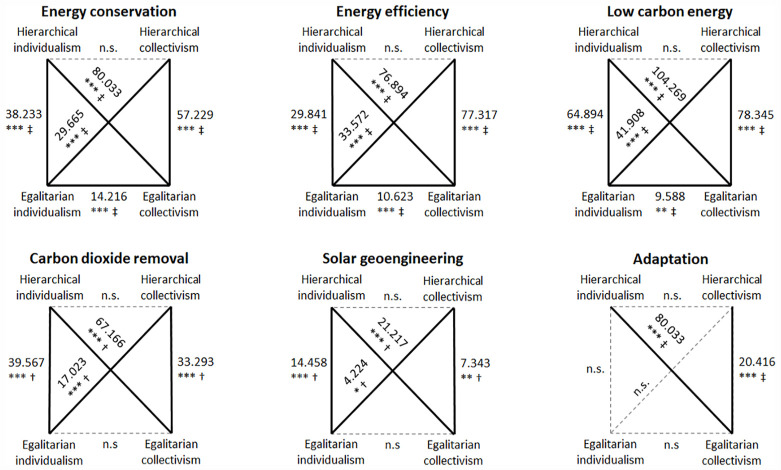
Differences in support for societal responses to climate tipping points between cultural worldviews. Bold lines signify statistically significant differences between cultural worldviews connected by the corresponding lines, while faint dotted lines signify non-significant differences between cultural worldviews connected by the corresponding lines. For example, under energy conservation, we can see significant differences between all cultural worldviews as shown by the bold connecting lines with corresponding test statistics, except between hierarchical individualism and hierarchical collectivism as shown by the faint dotted line between them. n.s.: not significant. *p < .05, **p < .01, ***p < .001. ^†^Dunn’s pairwise comparison post hoc test. ^‡^Pearson’s χ^2^ post hoc test.

Highly statistically significant differences were found between cultural worldviews for each of the different options under consideration – energy conservation (χ^2^(3, 1592) = 117.675, *p* < .001), energy efficiency (χ^2^(3, 1597) = 116.834, *p* < .001), low carbon energy (χ^2^(3, 1576) = 144.221, *p* < .001), carbon dioxide removal (*H*(3) = 85.290, *p* < .001), solar geoengineering (*H*(3) = 25.973, *p* < .001) and adaptation (χ^2^(3, 1469) = 29.506, *p* < .001). A series of Dunn’s pairwise comparisons and Pearson’s χ^2^ post hoc tests showed between which cultural worldviews the differences lay following the Kruskal–Wallis *H* tests and the Mood’s median tests, respectively. Figure 5 shows that those with an egalitarian worldview – either individualist or collectivist – are significantly more supportive of all six societal responses to climate tipping points than those with a hierarchical worldview, with the exception of adaptation where only egalitarian collectivists are significantly more supportive. In addition, egalitarian collectivists are significantly more supportive of energy conservation, energy efficiency and low carbon energy than egalitarian individualists.

Given the opportunity to explain their support or opposition to the option they felt most strongly about, most respondents selected energy efficiency (27.2%), energy conservation (23.0%) and low carbon energy (17.8%). Relatively fewer respondents selected carbon dioxide removal (13.0%), adaptation (10.3%) and solar geoengineering (8.8%). Thematic analysis of the responses revealed a wide variety of reasonings in support of and opposition to each option across the four cultural worldviews (see Supplemental Material).

Energy conservation was commonly supported for its reduction of waste and accessibility as something everyone could do, as well as its capacity to lower energy bills. It was also supported by those with a collectivist worldview for being counter-consumerism. However, it was criticised by hierarchical individualists for restricting people’s freedoms, and for self-sacrifice while other countries produced greater emissions.

Energy efficiency was commonly supported for its ease of implementation, reduction of waste, large scope for efficiencies to be found and its capacity to help save people money. It was also supported by those with an egalitarian worldview for placing the responsibility for action on producers. On the other hand, it was criticised by hierarchical individualists as a cynical money-making project.

Low carbon energy was commonly supported for it already being underway and ending dependence on fossil fuels. Particular forms of low carbon energy were also supported, including nuclear energy by those with a hierarchical worldview, and solar and wind energy by egalitarian collectivists. However, it was criticised by hierarchical individualists as something that could only be implemented by the rich and as a national self-sacrifice while other countries produced greater emissions.

Carbon dioxide removal was commonly supported for it directly tackling the problem of carbon dioxide in the air, its ease of implementation (particularly in the form of tree planting) and the fact that not all emissions can be eliminated. It was also supported by those with an individualist worldview for its non-reliance on behaviour change. On the other hand, it was criticised by egalitarian collectivists for being a distraction from emissions reductions.

Solar geoengineering was commonly seen as an innovative idea that would cool an Earth warming too fast, but one that was far-fetched, that does not address the root causes of climate tipping points and that tampers with nature and poses significant risks.

Adaptation was commonly seen as something needed to prepare for impacts already being felt and those yet to come. It was supported by those with a hierarchical worldview for being something that humans have always done in the face of environmental change. However, it was criticised by those with an egalitarian worldview for its sense of giving up, not being possible to adapt to some climate tipping points, and its diversion of attention from emissions reductions.

## 4. Discussion and conclusion

Awareness of climate tipping points among the British public is low in general and lower than previously reported studies with more educated respondents (Bellamy and Hulme, 2011). This is echoed by low familiarity among climate negotiators and members of the non-governmental organisation (NGO) community (Milkoreit, 2019). Of particular note is the very low awareness of slowdown in the AMOC, despite its popularisation in cinema (albeit nearly 20 years prior) (Lowe et al., 2006). People were most aware of ice loss from Arctic sea ice, which is consistent with previous surveys reporting relatively high awareness of the ‘iconic’ ecosystem (Gelcich et al., 2014; Scheffer et al., 2015). Consistent with previous research, people with hierarchical worldviews were significantly less aware of climate tipping points (Bellamy and Hulme, 2011). Women, younger, less educated and lower social grade respondents were also significantly less aware, although other research with sociological and socio-economic variables such as gender, age, education and social grade have often produced inconsistencies in the direction of relationships (McCright, 2009). Social media has shown promise for increasing the awareness of climate change in general (Mavrodieva et al., 2019), and with no relationship between the use of social media and awareness of climate tipping points found here, its potential appears so far untapped. Other possibilities include serious gaming, which has been shown to reduce the psychological distance of climate tipping points and provide effective science-policy engagement tools (Van Beek et al., 2022).

The British public is highly doubtful about the future effectiveness of humanity’s response to climate change in general. This is reflective of a tendency for news media to frame climate change in terms of inaction and consequences (Chen et al., 2022). What is more, the public are significantly more doubtful about humanity’s response to climate tipping points specifically. This contrasts with recent research showing no difference between perceptions of linear and nonlinear climate changes (Formanski et al., 2022), and is consistent with previous research showing that climate tipping points can instil a sense of fatalism (Bellamy and Hulme, 2011). While fearful representations of climate change are good at attracting attention – as illustrated by the rapidly growing invocation of climate tipping points in print and online media shown in Figure 2 – they are nevertheless an ineffective tool for motivating genuine personal engagement with climate change (Crucifix and Annan, 2019; O’Neill and Nicholson-Cole, 2009). The one exception to this is likely to be people with egalitarian worldviews, of whom, in confirmation of earlier research, significantly more judge climate tipping points likely to be crossed and to be a significant threat to humanity (Bellamy and Hulme, 2011). Significantly more women also judge climate tipping points to be a risk – a finding that is consistent with other research on climate change in general, but which is a weaker predictor of perception than worldview (Whitmarsh and Capstick, 2018).

Public doubt over the future effectiveness of humanity’s response to climate change begs the question as to what might be done about it. If communicative frames of inaction, consequences and climate tipping points lead to ‘apocalypse fatigue’ (Stoknes, 2015) and are at best only likely to motivate a genuine personal engagement with climate change among egalitarians, then new, more culturally sensitive ways of engaging will be needed to avoid polarisation (Kahan et al., 2012). This may include, for example, highlighting more hierarchical and individualistic values at risk from climate tipping points, or, more positively, societal responses that resonate better with such values.

On aggregate, public risk perceptions of climate tipping points deviate considerably from expert judgements (cf. Lenton, 2011). For the public, the dieback of the Amazon rainforest, ice loss from Arctic sea ice and ice loss from the Greenland ice sheet are most thought to be likely and threatening. For experts, while the Greenland ice sheet is thought to be a relatively high-probability, high-impact event, Arctic sea ice is thought to be a high-probability, low-impact event, and the Amazon is thought to be of a moderate probability and impact. For the public, ice loss from the West Antarctic ice sheet and dieback of boreal forest are both moderately thought to be likely and threatening. For experts, on the other hand, the West Antarctic ice sheet is thought to be a moderate-likelihood, high-impact event, and boreal forest is thought to be a low-probability, low-impact event. Finally, for the public, a strengthening of El Niño and a slowdown of the AMOC are the least thought to be likely and threatening. For experts, on the other hand, El Niño is thought to be a relatively low-probability, high-impact event, and the AMOC is thought to be a low-probability, moderate-impact event. The tipping points viewed as highest risk by the public appear to those iconic ecosystems with which they are most familiar, indicating further need for (culturally sensitive) communications.

In relation to preferences for societal responses to climate tipping points, both energy conservation and energy efficiency enjoy strong public support, and echoing findings elsewhere in the literature, this is nevertheless dependent on conditions such as fairness and trust being met (Bellamy et al., 2016; Cherry et al., 2018). Support for low carbon energy was also high, although again subject to conditions such as fairness, and with particular forms of energy often expressly preferred in ways that reflect known cultural biases (Kahan, 2012). Support for carbon dioxide removal often linked to tree planting, reflecting a well-documented preference for ostensibly ‘natural’ approaches (Bellamy, 2022), and concerns about distracting from emissions reductions echo common viewpoints also found in the literature (Cox et al., 2020). Concerns around solar geoengineering, mainly around its unintended consequences, also confirm those in other studies (Bellamy et al., 2016). Support for adaptation was only slightly higher than that for solar geoengineering, reflecting long-standing – if not entirely justified – criticisms about limits and distracting from emissions reductions (Pielke et al., 2007). The strongest support for societal responses to climate tipping points overall comes from those with egalitarian values, but crucially, all options received great support from the public as a whole. Contrary to recent calls to disincentivise certain areas of climate solutions research (Biermann et al., 2022), this shows a public mandate for researching, if not necessarily deploying, all available options.

This article has revealed a number of significant results concerning how the British public perceives the risks of climate tipping points and their preferences in responding to them. These results do not come without limitations, however. The methods rely on self-reported measures, which are relatively efficient and inexpensive to collect. These recognise that people are the best-qualified witnesses to their own perspectives; that people are motivated to think about themselves; and that there is a strong causal force between people’s self-perceptions and how they interact with the world (Paulhus and Vazire, 2009). However, the validity of self-reports can be limited by socially desirable responding, acquiescent responding and extreme responding. In addition, self-knowledge may be constrained by an inability to recall all information relevant to a posed question. The cultural cognition thesis is also not without criticism, including that empirical testing has been largely limited to the United States (Van der Linden, 2015). Although it has also been successfully used in England (see Kahan et al., 2015), questions remain about its applicability to other countries.

In response to the threat of climate tipping points, there are growing calls for research and enactment of ‘social tipping points’ towards positive climate action (Milkoreit et al., 2018; Winkelmann et al., 2022). Proposed social tipping elements include social norms (Nyborg et al., 2016), agent capacities (Tàbara et al., 2018), policy interventions and governance (Otto et al., 2020; Sharpe and Lenton, 2020) and enterprise and informal peer enforcement (Otto et al., 2020). This article shows that if we are to understand or encourage such social tipping points, we must first contend with four fundamentally opposing cultural worldviews. Cultural theory holds that in any complex social system, a minimum requisite variety of these four worldviews will always be present (Thompson et al., 1990): ‘conflict among cultures is a precondition of cultural identity’ (Wildavsky, 1987: 7). Prospective social tipping points must therefore work with these worldviews, rather than against them. In other words, social tipping points should seek to tip support for climate policies not by trying to get people who think differently to *think* the same thing, but by getting people who think differently to *do* the same thing. It is not about tipping people from being hierarchical individualists to being egalitarian collectivists, for example; it is about designing and/or communicating climate policies in ways that garner support from both social groups. One way of doing this would be to design or facilitate ‘clumsy’ solutions that incorporate responses to climate change that different worldviews can get behind (Verweij et al., 2006). Another way would be to identify what we might call ‘cultural tipping elements’ in the way we communicate the same response to climate change to different worldviews. By emphasising key values of interest that stand to benefit from a given climate action and de-emphasising others, we could find creative ways of building support for the same things, but for different reasons.

## Supplemental Material

sj-docx-1-pus-10.1177_09636625231177820 – Supplemental material for Public perceptions of climate tipping points Click here for additional data file. Supplemental material, sj-docx-1-pus-10.1177_09636625231177820 for Public perceptions of climate tipping points by Rob Bellamy in Public Understanding of Science

## References

[bibr1-09636625231177820] Antilla L (2010) Self-censorship and science: A geographical review of media coverage of climate tipping points. Public Understanding of Science 19: 240–256.

[bibr2-09636625231177820] Bellamy R (2022) Mapping public appraisals of carbon dioxide removal. Global Environmental Change 76: 102593.

[bibr3-09636625231177820] Bellamy R Hulme M (2011) Beyond the tipping point: Understanding perceptions of abrupt climate change and their implications. Weather, Climate, and Society 3: 48–60.

[bibr4-09636625231177820] Bellamy R Chilvers J Vaughan N (2016) Deliberative mapping of options for tackling climate change: Citizens and specialists ‘open up’ appraisal of geoengineering. Public Understanding of Science 25: 269–286. 25224904 10.1177/0963662514548628 PMC4819797

[bibr5-09636625231177820] Biermann F Oomen J Gupta A Ali S Conca K Hajer M , et al. (2022) Solar geoengineering: The case for an international non-use agreement. WIREs Climate Change 13: e754.

[bibr6-09636625231177820] Braun V Clarke V (2006) Using thematic analysis in psychology. Qualitative Research in Psychology 3: 77–101.

[bibr7-09636625231177820] Caesar L Rahmstorf S Robinson A Feulner G Saba V (2018) Observed fingerprint of a weakening Atlantic Ocean overturning circulation. Nature 556: 191–196. 29643485 10.1038/s41586-018-0006-5

[bibr8-09636625231177820] Caldeira K Bala G Cao L (2013) The science of geoengineering. The Annual Review of Earth and Planetary Sciences 41: 231–256.

[bibr9-09636625231177820] Chen K Molder A Duan Z Boulianne S Eckart C Mallari P , et al. (2022) How climate movement actors and news media frame climate change and strike: Evidence from analysing Twitter and news media discourse from 2018 to 2021. The International Journal of Press/Politics 28: 384–413.

[bibr10-09636625231177820] Cherry C Scott K Barrett J Pidgeon N (2018) Public acceptance of resource-efficiency strategies to mitigate climate change. Nature Climate Change 8: 1007–1012.

[bibr11-09636625231177820] Cox E Spence E Pidgeon N (2020) Public perceptions of carbon dioxide removal in the United States and the United Kingdom. Nature Climate Change 10: 744–749.

[bibr12-09636625231177820] Crucifix M Annan J (2019) Is the concept of ‘tipping point’ helpful for describing and communicating possible climate futures? In: Hulme M (ed.) Contemporary Climate Change Debates. London and New York: Routledge, pp. 23–35.

[bibr13-09636625231177820] Douglas M Wildavsky A (1982) Risk and Culture: An Essay on the Selection of Technological and Environmental Dangers. Berkeley, CA: University of California Press.

[bibr14-09636625231177820] Feldmann J Levermann A (2015) Collapse of the West Antarctic Ice Sheet after local destabilization of the Amundsen Basin. Proceedings of the National Academy of Sciences of the United States of America 112: 14191–14196. 26578762 10.1073/pnas.1512482112 PMC4655561

[bibr15-09636625231177820] Formanski F Pein M Loschelder D Engler J Husen O Majer J (2022) Tipping points ahead? How laypeople respond to linear versus nonlinear climate change predictions. Climatic Change 175: 8. 36439364 10.1007/s10584-022-03459-z PMC9676726

[bibr16-09636625231177820] Gelcich S Buckley P Pinnegar J Chilvers J Lorenzoni I Terry G , et al. (2014) Public awareness, concerns, and priorities about anthropogenic impacts on marine environments. Proceedings of the National Academy of Sciences of the United States of America 111: 15042–15047. 25288740 10.1073/pnas.1417344111 PMC4210304

[bibr17-09636625231177820] IPCC (2021) Climate Change 2021: The Physical Science Basis. Contribution of Working Group I to the Sixth Assessment Report of the Intergovernmental Panel on Climate Change, IPCC.

[bibr18-09636625231177820] Jackson L Kahana R Graham T Ringer M Woollings T Mecking J , et al. (2015) Global and European climate impacts of a slowdown of the AMOC in a high resolution GCM. Climate Dynamics 45: 3299–3316.

[bibr19-09636625231177820] Kahan D (2012) Cultural cognition as a conception of the cultural theory of risk. In: Roeser S Hillerbrand R Sandin P Peterson M (eds.) Handbook of Risk Theory: Epistemology, Decision Theory, Ethics, and Social Implications of Risk. London: Springer, pp. 725–759.

[bibr20-09636625231177820] Kahan D Jenkins-Smith H Tarantola T Silva C Braman D (2015) Geoengineering and climate change polarization: Testing a two-channel model of science communication. The Annals of the American Academy of Political and Social Science 658: 192–222.

[bibr21-09636625231177820] Kahan D Peters E Wittlin M Slovic P Ouellette L Braman D , et al. (2012) The polarizing impact of science literacy and numeracy on perceived climate change risks. Nature Climate Change 2: 732–735.

[bibr22-09636625231177820] Kemp L Xu C Depledge J Ebi K Gibbins G Kohler T , et al. (2022) Climate endgame: Exploring catastrophic climate change scenarios. Proceedings of the National Academy of Sciences of the United States of America 119: e2108146119. 10.1073/pnas.2108146119 PMC9407216 35914185

[bibr23-09636625231177820] Lenton T (2011) Early warning of climate tipping points. Nature Climate Change 1: 201–209.

[bibr24-09636625231177820] Lenton T Held H Kriegler E Hall J Lucht W Rahmstorf S , et al. (2008) Tipping elements in the Earth’s climate system. Proceedings of the National Academy of Sciences of the United States of America 105: 1786–1793. 18258748 10.1073/pnas.0705414105 PMC2538841

[bibr25-09636625231177820] Lenton T Rockström J Gaffney O Rahmstorf S Richardson K Steffen W , et al. (2019) Climate tipping points – too risky to bet against. Nature 575: 592–595. 31776487 10.1038/d41586-019-03595-0

[bibr26-09636625231177820] Lowe T Brown K Dessai S França Doria M Haynes K Vincent K (2006) Does tomorrow ever come? Disaster narrative and public perceptions of climate change. Public Understanding of Science 15: 435–457.

[bibr27-09636625231177820] McCright A (2009) The social bases of climate change knowledge, concern, and policy support in the U.S. General Public. Hofstra Law Review 37: 1017–1046.

[bibr28-09636625231177820] McKay D Staal A Abrams J Winkelmann R Sakschewski B Loriani S , et al. (2022) Exceeding 1.5°C global warming could trigger multiple climate tipping points. Science 377: 1–10. 10.1126/science.abn7950 36074831

[bibr29-09636625231177820] Mavrodieva A Rachman O Harahap V Shaw R (2019) Role of social media as a soft power tool in raising public awareness and engagement in addressing climate change. Climate 7: 122.

[bibr30-09636625231177820] Mengel M Levermann A (2014) Ice plug prevents irreversible discharge from East Antarctica. Nature Climate Change 4: 451–455.

[bibr31-09636625231177820] Milkoreit M (2019) Cognitive capacities for global governance in the face of complexity: The case of climate tipping points. In: Galaz V (ed.) Global Challenges, Governance, and Complexity: Applications and Frontiers. Cheltenham: Edward Elgar Publishing, pp. 274–302.

[bibr32-09636625231177820] Milkoreit M Hodbod J Baggio J Benessaiah K Calderón -Contreras R Donges J , et al. (2018) Defining tipping points for social-ecological systems scholarship – An interdisciplinary literature review. Environmental Research Letters 13: 033005.

[bibr33-09636625231177820] Nyborg K Anderies J Dannenberg A Lindahl T Schill C Schlüter M , et al. (2016) Social norms as solutions: Policies may influence large-scale behavioral tipping. Science 354: 42–43. 27846488 10.1126/science.aaf8317

[bibr34-09636625231177820] O’Neill S Nicholson-Cole S (2009) ‘Fear won’t do it’: Promoting positive engagement with climate change through visual and iconic representations. Science Communication 30: 355–379.

[bibr35-09636625231177820] Otto I Donges J Cremades R Schellnhuber H (2020) Social tipping dynamics for stabilizing Earth’s climate by 2050. Proceedings of the National Academy of Sciences of the United States of America 117: 2354–2365. 31964839 10.1073/pnas.1900577117 PMC7007533

[bibr36-09636625231177820] Paulhus D Vazire S (2009) The self-report method. In: Robins R Fraley C Krueger R (eds) Handbook of Research Methods in Personality Psychology. New York, NY: The Guilford Press, pp. 224–239.

[bibr37-09636625231177820] Pielke R Prins G Rayner S Sarewitz D (2007) Lifting the taboo on adaptation. Nature 445: 597–598. 17287795 10.1038/445597a

[bibr38-09636625231177820] Rocha J Peterson G Bodin O Levin S (2018) Cascading regime shifts within and across scales. Science 362: 1379–1383. 30573623 10.1126/science.aat7850

[bibr39-09636625231177820] Russill C Nyssa Z (2009) The tipping point trend in climate change communication. Global Environmental Change 19: 336–344.

[bibr40-09636625231177820] Scheffer M Barrett S Carpenter S Folke C Green A Holmgren M , et al. (2015) Creating a safe operating space for iconic ecosystems. Science 347: 1317–1319. 25792318 10.1126/science.aaa3769

[bibr41-09636625231177820] Sharpe S Lenton T (2020) Upward-scaling tipping cascades to meet climate goals: Plausible grounds for hope. Climate Policy 21: 421–433.

[bibr42-09636625231177820] Steffen W Rockström J Richardson K Lenton T Folke C Liverman D , et al. (2018) Trajectories of the Earth system in the Anthropocene. Proceedings of the National Academy of Sciences of the United States of America 115: 8252–8259. 30082409 10.1073/pnas.1810141115 PMC6099852

[bibr43-09636625231177820] Stoknes P (2015) What we Think About When we try not to Think About Global Warming: Toward a New Psychology of Climate Action. Chelsea, VT: Chelsea Green Publishing.

[bibr44-09636625231177820] Tàbara J Frantzeskaki N Hölscher K Pedde S Kok K Lamperti F , et al. (2018) Positive tipping points in a rapidly warming world. Current Opinion in Environmental Sustainability 31: 120–129.

[bibr45-09636625231177820] Thompson M Ellis R Wildavsky A (1990) Cultural Theory. Oxon: Routledge.

[bibr46-09636625231177820] Van Beek L Milkoreit M Prokopy L Reed J Vervoort J Wardekker A , et al. (2022) The effects of serious gaming on risk perceptions of climate tipping points. Climatic Change 170: 31.

[bibr47-09636625231177820] Van der Hel S Hellsten I Steen G (2018) Tipping points and climate change: Metaphor between science and the media. Environmental Communication 12: 605–620.

[bibr48-09636625231177820] Van der Linden S (2015) A conceptual critique of the cultural cognition thesis. Science Communication 38: 128–138.

[bibr49-09636625231177820] Verweij M Douglas M Ellis R Engel C Henriks F Lohmann S , et al. (2006) Clumsy solutions for a complex world: The case of climate change. Public Administration 84: 817–843.

[bibr50-09636625231177820] Whitmarsh L Capstick S (2018) Perceptions of climate change. In: Clayton S Manning C (eds) Psychology and Climate Change: Human Perceptions, Impacts, and Responses. London: Elsevier Academic Press, pp. 13–33.

[bibr51-09636625231177820] Wildavsky A (1987) Choosing preferences by constructing institutions: A cultural theory of preference formation. American Political Science Review 81: 3–21.

[bibr52-09636625231177820] Winkelmann R Donges J Smith E Milkoreit M Eder C Heitzig J , et al. (2022) Social tipping processes towards climate action: A conceptual framework. Ecological Economics 192: 107242.

