# Supplementary material for: Public perceptions of climate tipping points
Source: Public Underst Sci. 2023 Jun 28;32(8):1033–47. doi: 10.1177/09636625231177820 (PMC10631267; doi:10.1177/09636625231177820)
Supplement: sj-docx-1-pus-10.1177_09636625231177820 – Supplemental material for Public perceptions of climate tipping points [file sj-docx-1-pus-10.1177_09636625231177820.docx]

**Public perceptions of climate tipping points**

Rob Bellamy^1^*

^1^ University of Manchester, Department of Geography, Manchester, UK

* rob.bellamy@manchester.ac.uk

**Supplemental Material**

**Table of contents**

1. Sample attributes

2. Survey screen captures

3. Cultural cognition map of survey respondents’ worldviews

4. Pearson’s χ^2^ tests of difference between cultural worldviews, genders, ages, levels of education and social grades in relation to awareness, perceived likelihood and perceived threat of climate tipping points

5. Thematic analysis of reasonings underpinning support and opposition to societal responses to climate tipping points between cultural worldviews

**1. Sample attributes**

| **Attribute** | **Variable** | **Percent of sample^†^** | **Percent of population^‡^** |
| --- | --- | --- | --- |
| Age | 18-24 | 8.5 | 11.1 |
|  | 25-49 | 40.5 | 42.0 |
|  | 50-65 | 26.9 | 23.8 |
|  | Over 65 | 24.1 | 23.1 |
| Gender | Male | 44.3 | 48.4 |
|  | Female | 55.7 | 51.6 |
| Social grade^‖^ | AB | 32.0 | 28.0 |
|  | C1 | 28.1 | 29.0 |
|  | C2 | 17.1 | 21.0 |
|  | DE | 22.8 | 22.0 |
| Region | South | 23.7 | 32.6 |
|  | London | 10.1 | 11.7 |
|  | Midlands | 26.0 | 16.0 |
|  | North | 23.2 | 23.7 |
|  | Wales | 5.3 | 4.9 |
|  | Scotland | 8.5 | 8.5 |
|  | Northern Ireland | 3.2 | 2.6 |
| 2019 election vote^§^ | Conservative | 32.8 | 43.6 |
|  | Labour | 23.8 | 32.1 |
|  | Liberal Democrat | 8.5 | 11.6 |
| EU referendum vote^§^ | Remain | 41.1 | 38.6 |
|  | Leave | 37.8 | 40.8 |
| Cultural worldview | Egalitarian individualism | 27.2 | N/A |
|  | Hierarchical individualism | 21.7 | N/A |
|  | Hierarchical collectivism | 15.2 | N/A |
|  | Egalitarian collectivism | 33.4 | N/A |

† Percentages may not add up to 100 due to rounding.

‡ As weighted by the specialist panel company according to 2011 census data, large-scale random probability surveys, 2019 general election and 2016 referendum results, and official ONS population estimates.

§ Percentages do not add up to 100 due to other votes, no votes or non-responses.

‖ Socio-economic classification produced by the UK Office for National Statistics where AB denotes higher & intermediate managerial, administrative, professional occupations; C1 denotes supervisory, clerical & junior managerial, administrative, professional occupations; C2 denotes skilled manual occupations; and DE denotes semi-skilled & unskilled manual occupations, Unemployed and lowest grade occupations.

**2. Survey screen captures**

Page 1:


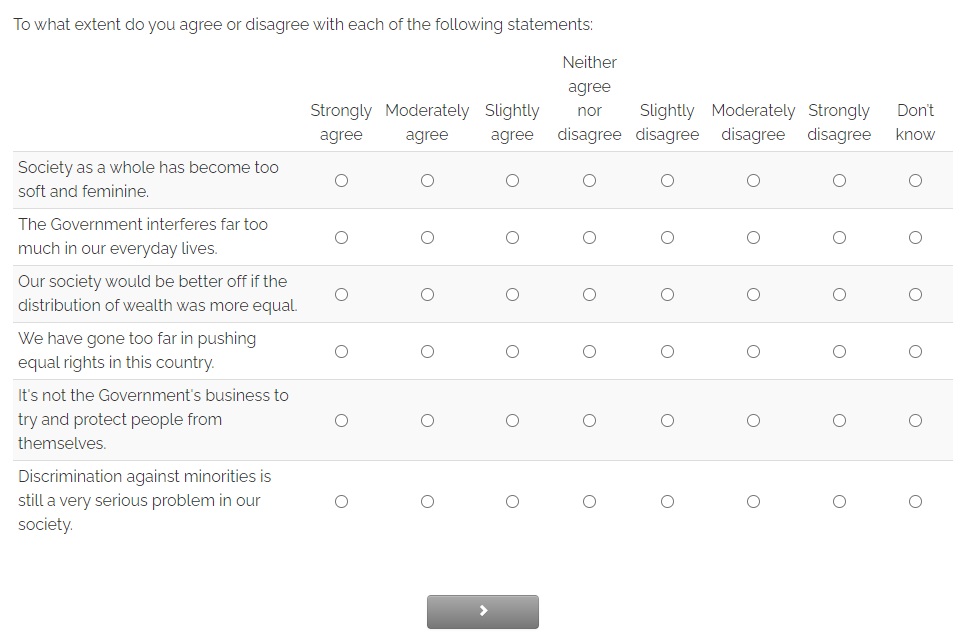


Page 2:


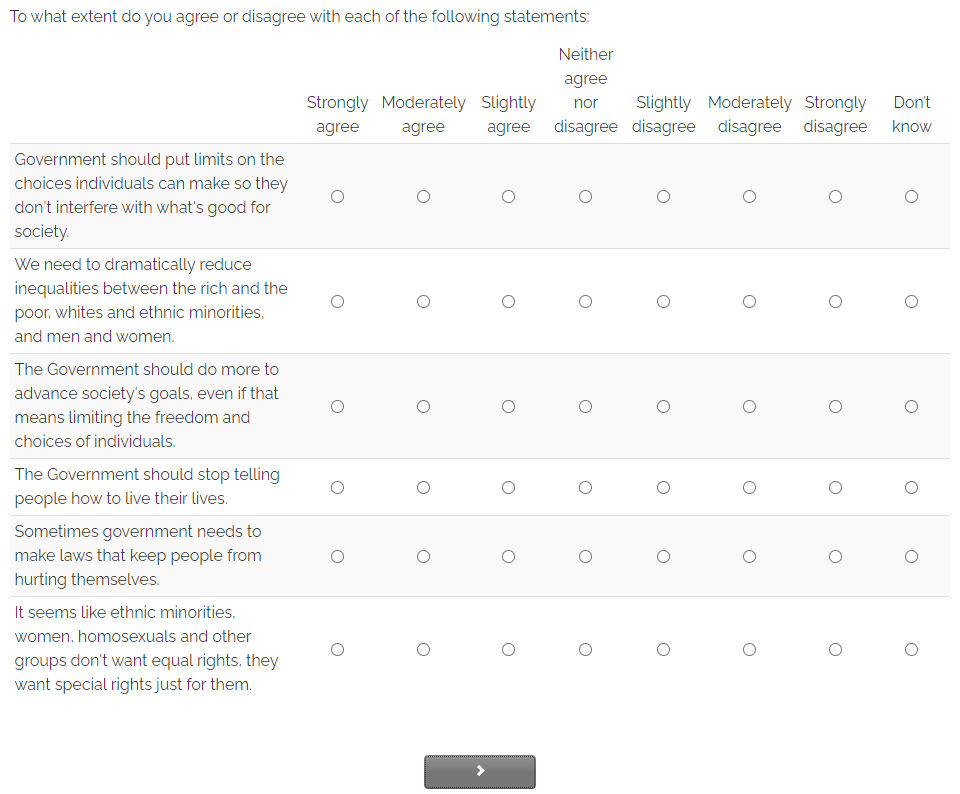


Page 3:


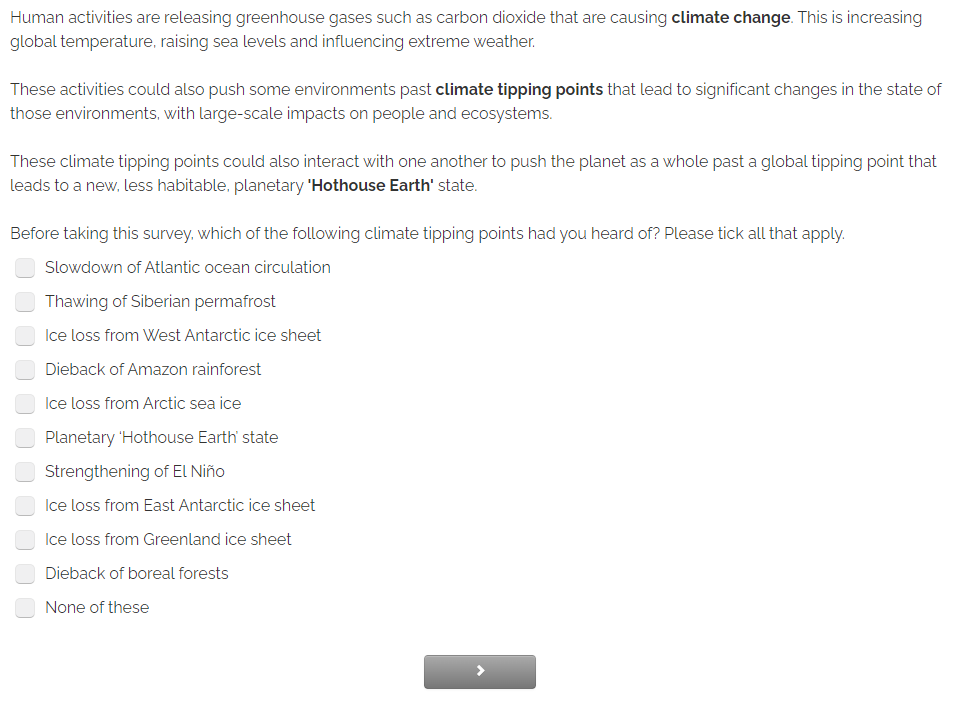


Page 4:


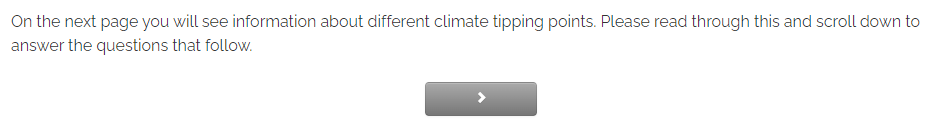


Page 5:


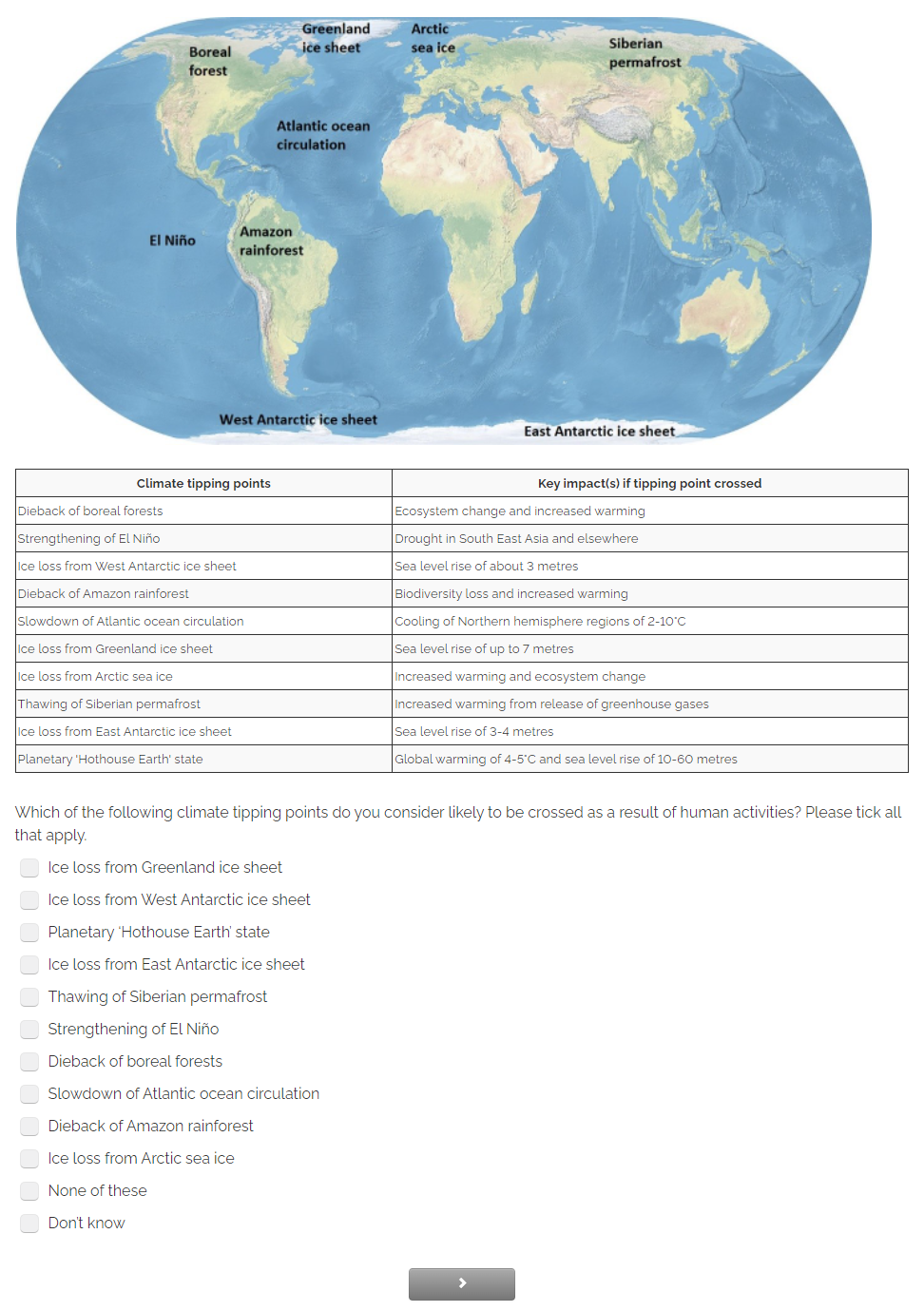


Page 6:


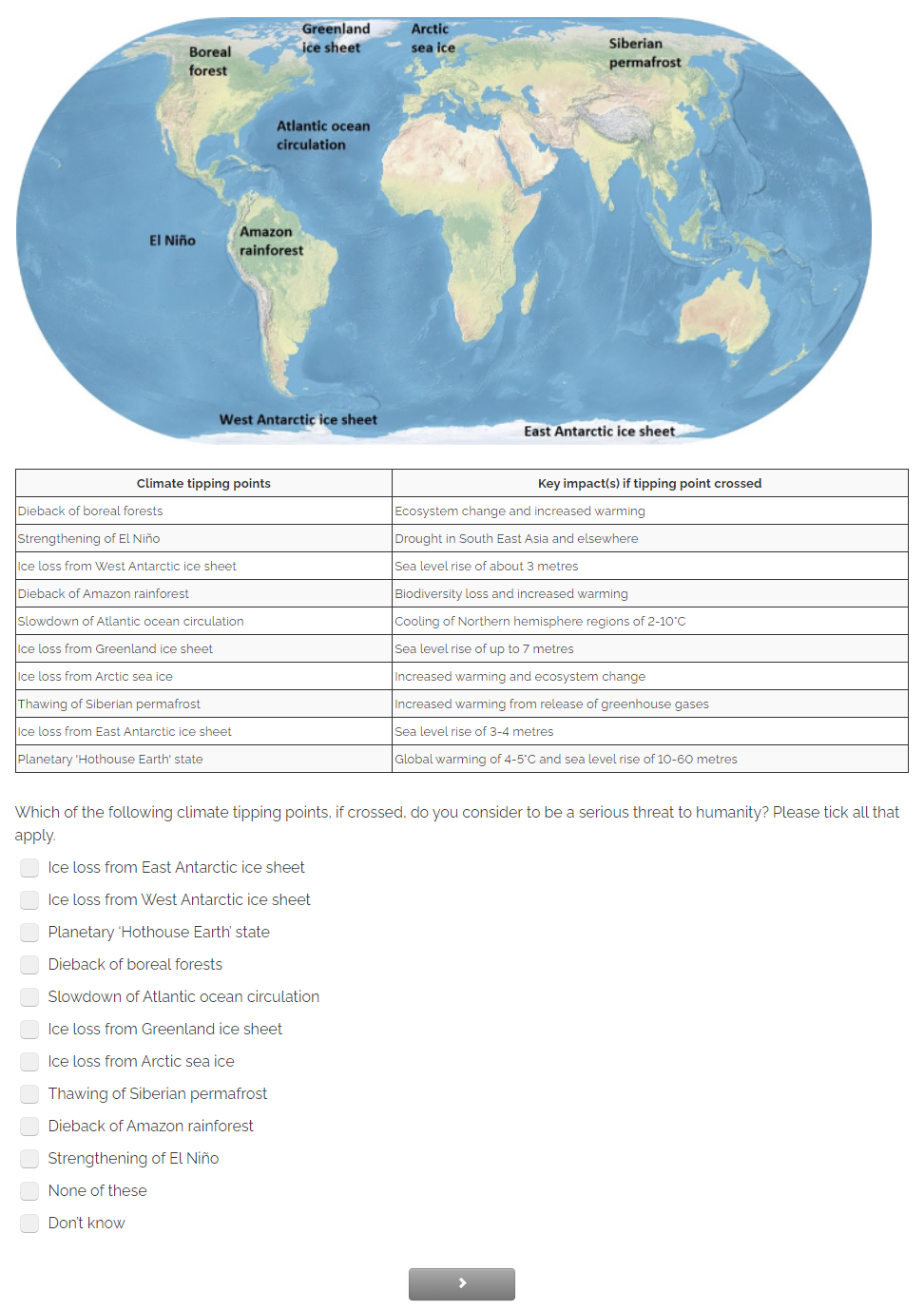


Page 7:


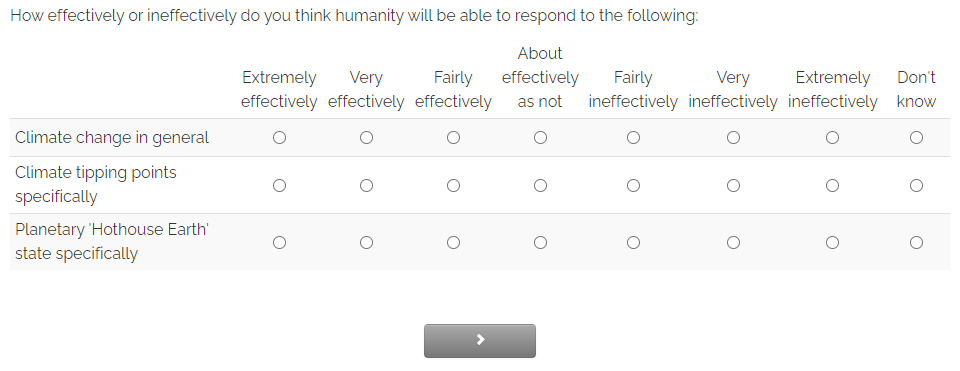


Page 8:


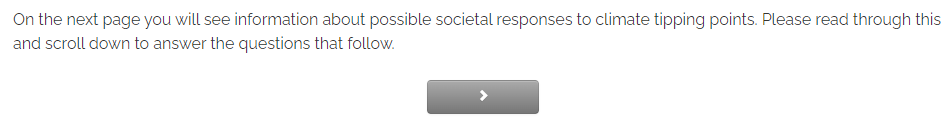


Page 9:


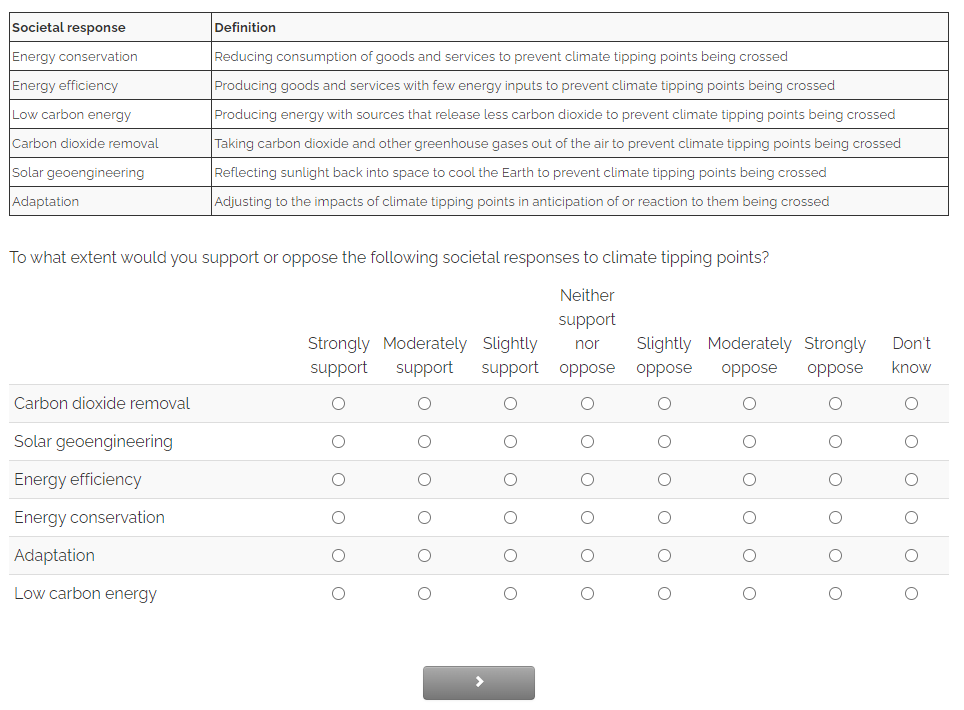


Page 10:


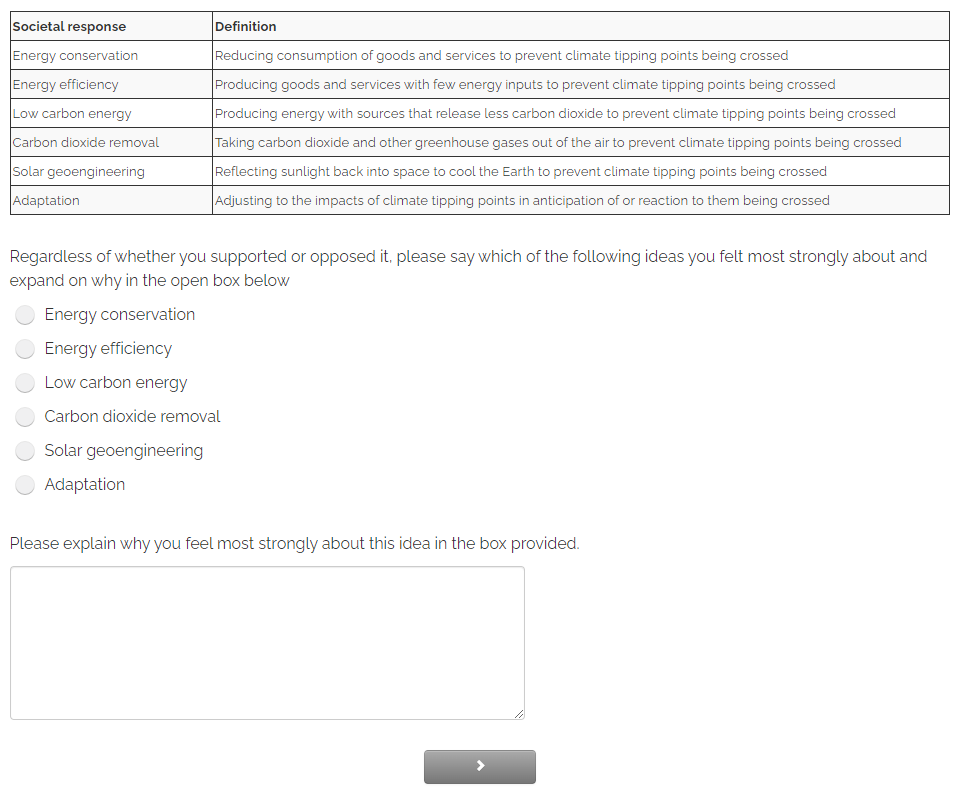


**3. Cultural cognition map of survey respondents’ worldviews**


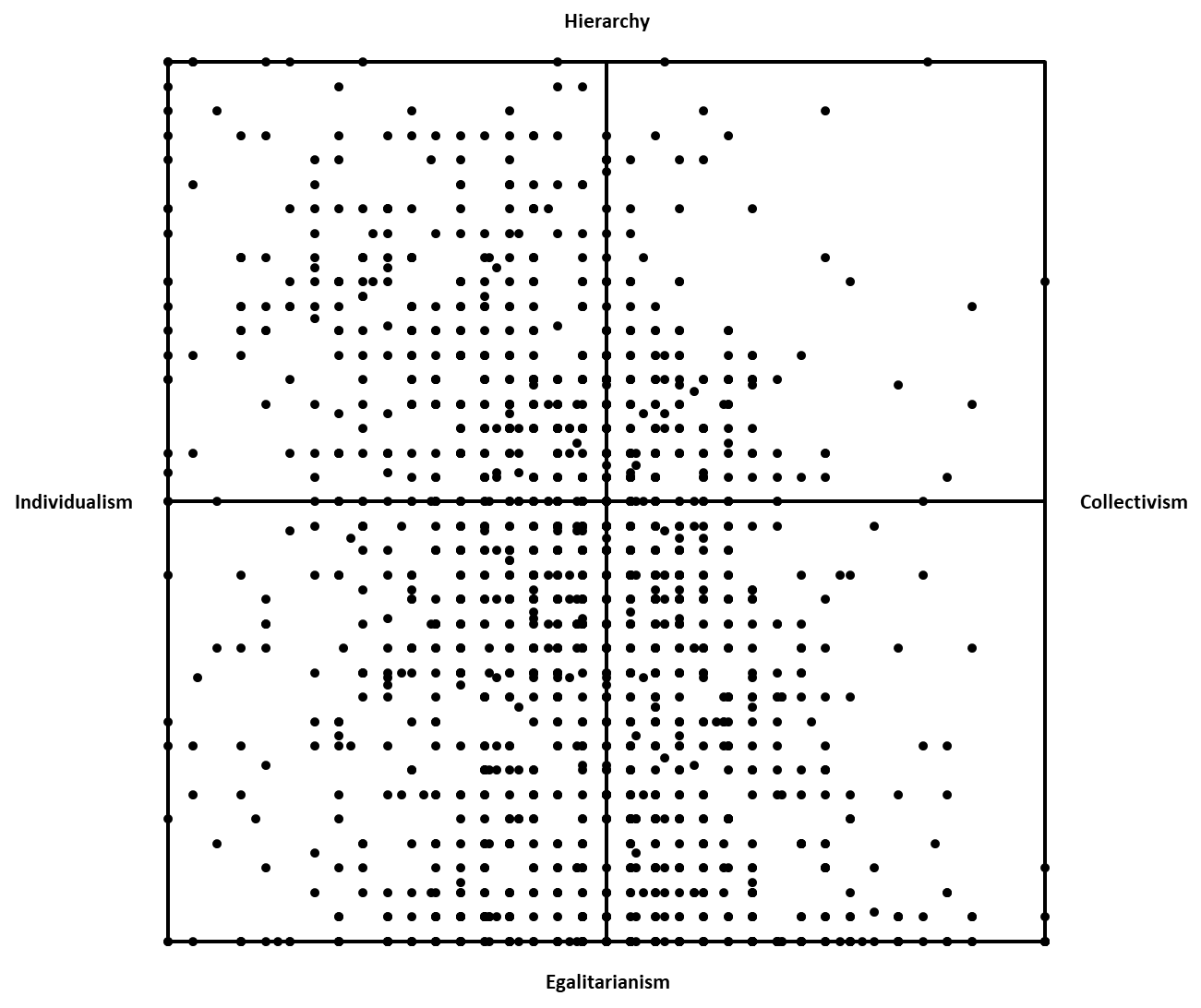


**4. Pearson’s χ^2^ tests of difference between cultural worldviews, genders, ages, levels of education and social grades in relation to awareness, perceived likelihood and perceived threat of climate tipping points**

|  | **Egalitarian individualism** | **Hierarchical individualism** | **Hierarchical collectivism** | **Egalitarian collectivism** |
| --- | --- | --- | --- | --- |
| **Awareness** | n.s. | n.s. | n.s. | χ2 (1, 1,731) = 8.221, p < .01 (>) |
| **Perceived likelihood** | χ2 (1, 1,731) = 11.573, p < .001 (>) | χ2 (1, 1,731) = 69.169, p < .001 (<) | n.s. | χ2 (1, 1,731) = 26.877, p < .001 (>) |
| **Perceived threat** | χ2 (1, 1,731) = 5.808, p < .001 (>) | χ2 (1, 1,731) = 56.466, p < .001 (<) | n.s. | χ2 (1, 1,731) = 20.081, p < .001 (>) |

|  | **Male** | **Female** |
| --- | --- | --- |
| **Awareness** | χ2 (1, 1,773) = 6.518, p < .05 (>) | n.s. |
| **Perceived likelihood** | n.s. | χ2 (1, 1,773) = 7.659, p < .01 (>) |
| **Perceived threat** | n.s. | χ2 (1, 1,773) = 11.826, p < .001 (>) |

|  | **18-24** | **25-34** | **35-44** | **45-54** | **55-64** | **65-74** | **75+** |
| --- | --- | --- | --- | --- | --- | --- | --- |
| **Awareness** | n.s. | χ2 (1, 1,733) = 15.601, p < .001 (<) | χ2 (1, 1,733) = 20.026, p < .001 (<) | χ2 (1, 1,733) = 3.926, p < .05 (<) | χ2 (1, 1,733) = 8.643, p < .01 (>) | χ2 (1, 1,733) = 29.508, p < .001 (>) | χ2 (1, 1,733) = 14.195, p < .001 (>) |
| **Perceived likelihood** | n.s. | n.s. | n.s. | n.s. | n.s. | n.s. | n.s. |
| **Perceived threat** | n.s. | n.s. | n.s. | n.s. | n.s. | n.s. | n.s. |

|  | **I** | **II** | **IIIb** | **IIIa** | **IV** | **V1** | **V2** |
| --- | --- | --- | --- | --- | --- | --- | --- |
| **Awareness** | χ2 (1, 1,733) = 13.686, p < .001 (<) | n.s. | n.s. | n.s. | n.s. | χ2 (1, 1,733) = 14.637, p < .001 (>) | χ2 (1, 1,733) = 17.727, p < .001 (>) |
| **Perceived likelihood** | χ2 (1, 1,773) = 18.545, p < .001 (<) | n.s. | n.s. | n.s. | n.s. | n.s. | χ2 (1, 1,773) = 4.159, p < .05 (>) |
| **Perceived threat** | n.s. | n.s. | n.s. | n.s. | n.s. | n.s. | n.s. |

|  | **A** | **B** | **C1** | **C2** | **D** | **E** |
| --- | --- | --- | --- | --- | --- | --- |
| **Awareness** | χ2 (1, 1,733) = 5.423, p < .05 (>) | χ2 (1, 1,733) = 7.377, p < .01 (>) | n.s. | n.s. | χ2 (1, 1,733) = 14.473, p < .001 (<) | n.s. |
| **Perceived likelihood** | n.s. | n.s. | n.s. | n.s. | n.s. | n.s. |
| **Perceived threat** | χ2 (1, 1,773) = 4.620, p < .05 (>) | n.s. | n.s. | n.s. | n.s. | n.s. |

n.s. = not significant. (>) significantly higher than the rest of the sample population, (<) significantly lower than the rest of the sample population.

**5. Thematic analysis of reasonings underpinning support and opposition to societal responses to climate tipping points between cultural worldviews**

|  | **Egalitarian individualists** | **Hierarchical individualists** | **Hierarchical collectivists** | **Egalitarian collectivists** |
| --- | --- | --- | --- | --- |
| **Energy conservation** | (+) Everyone can do it  (+) Need to reduce waste  (+) Co-benefits, e.g. bills  (+) Prevents problem  (=) Re-education needed | (+) Need to reduce waste  (=) Feasibility of option  (=) Cost of option  (-) Others emit more  (-) Restricting freedoms | (+) Big collective impact  (+) Prevents wasteful use  (+) Less consumerism  (+) Easy to implement  (+) Helps to save money | (+) Easy to implement  (+) Stop wastefulness  (+) Stop overconsumption  (+) Everyone can do it  (+) Counter consumerism |
| **Energy efficiency** | (+) Easy to implement  (+) Reduces waste  (+) Helps to save money  (+) Producers responsible  (+) Scope for efficiencies | (+) New product efficiency  (+) Easy to implement  (+) Stimulates innovation  (+) Lower energy prices  (-) Money-making project | (+) Easy to implement  (+) Helps to save money  (+) Carry on and modify  (+) Helps to save energy  (+) Scope for efficiencies | (+) Easy to implement  (+) Prevents wastage  (+) Onus on producers  (+) Helps to save money  (+) Little impact on society |
| **Low carbon energy** | (+) Ends fossil fuel use  (+) Consumption remains  (+) Maintains economy  (+) Energy independence  (=) Ease of action | (+) Already underway  (+) More nuclear power  (=) Fossil fuel alternative  (-) Inequalities in access  (-) Others do not act | (+) Use less fossil fuels  (+) Prevents pollution  (+) Every country can do it  (+) Need to accelerate  (+) Nuclear will be best | (+) Easy to implement  (+) More solar and wind  (+) Reduce fossil fuels  (+) Technology available  (+) No lifestyle changes |
| **Carbon dioxide removal** | (+) Innovative solution  (+) No behaviour change  (+) Tackles carbon directly  (+) Easy, e.g. tree planting  (+) Can reverse damage | (+) Things can carry on  (+) Removes carbon  (+) Offset some emissions  (+) Planting more trees  (-) Takes time to scale | (+) Tackles carbon directly  (+) Can plant lots of trees  (+) Offset some emissions  (=) Ease of action  (=) Safety of option | (+) Removes emissions  (+) Reduction not working  (+) Easy, e.g. new habitats  (=) Cleaner air for health  (-) Distraction and storage |
| **Solar geoengineering** | (+) Innovative idea  (+) Will cool Earth and ice  (=) Cost of option  (-) Does not address cause  (-) Scary last resort | (+) Cheap and actionable  (=) Novel but far-fetched  (-) Adverse side effects  (-) Does not address cause  (-) Large surface area | (+) Easy to implement  (+) Earth warming too fast  (+) All countries can help  (=) Novel but far-fetched  (-) Tampers with nature | (+) No change to lifestyles  (=) Effective but narrow  (=) Cost effective option  (-) Unforeseen impacts  (-) Undermines mitigation |
| **Adaptation** | (+) Need to be prepared  (+) Prevention not enough  (-) Selfish to vulnerable  (-) Feels like giving up  (-) Limits to adaptation | (+) Adjust to new world  (+) Collective action fails  (+) Have always adapted  (+) Often ignored option  (=) Prevention or cure | (+) Impacts already here  (+) Have always adapted  (+) Avoids ‘green’ energy  (+) Population control  (=) Autonomous but slow | (+) Need to be prepared  (+) Tipping points certain  (-) Adapting not possible  (-) Undermines mitigation  (-) Accepting capitalism |

Themes preceded by (+) indicate themes supportive of the option in question; those preceded by (-) indicate themes oppositional to the option in question; and those preceded by (=) indicate themes with both supportive of and oppositional to the option in question.
